# Ruthenium(II)–Cyclopentadienyl-Derived Complexes as New Emerging Anti-Colorectal Cancer Drugs

**DOI:** 10.3390/pharmaceutics14061293

**Published:** 2022-06-17

**Authors:** Catarina Teixeira-Guedes, Ana Rita Brás, Ricardo G. Teixeira, Andreia Valente, Ana Preto

**Affiliations:** 1 Centre of Molecular and Environmental Biology (CBMA), Department of Biology, University of Minho, Campus de Gualtar, 4710-057 Braga, Portugal; cigteixeira@gmail.com (C.T.-G.); pg31015@alunos.uminho.pt (A.R.B.); 2 Institute of Science and Innovation for Bio-Sustainability (IB-S), University of Minho, Campus de Gualtar, 4710-057 Braga, Portugal; 3 Centre for the Research and Technology of Agro-Environmental and Biological Sciences (CITAB), University of Trás dos Montes and Alto Douro, Quinta de Prados, 5000-801 Vila Real, Portugal; 4 Centro de Química Estrutural, Institute of Molecular Sciences and Departamento de Química e Bioquímica, Faculdade de Ciências, Universidade de Lisboa, Campo Grande, 1749-016 Lisboa, Portugal; rjteixeira@fc.ul.pt

**Keywords:** ruthenium complexes, anti-colorectal cancer drugs, apoptosis, cell cycle arrest, cytoskeleton

## Abstract

Colorectal cancer (CRC) is one of the most common malignancies and one of the leading causes of cancer-related death worldwide, urging the need for new and more efficient therapeutic approaches. Ruthenium complexes have emerged as attractive alternatives to traditional platinum-based compounds in the treatment of CRC. This work aims to evaluate anti-CRC properties, as well as to identify the mechanisms of action of ruthenium complexes with the general formula [Ru(η^5^-C_5_H_4_R)(PPh_3_)(4,4′-R′-2,2′-bipyridine)][CF_3_SO_3_], where R = CH_3_, CHO or CH_2_OH and R′ = H, CH_3_, CH_2_OH, or dibiotin ester. The complexes (Ru 1–7) displayed high bioactivity, as shown by low IC_50_ concentrations against CRC cells, namely, RKO and SW480. Four of the most promising ruthenium complexes (Ru 2, 5–7) were phenotypically characterized and were shown to inhibit cell viability by decreasing cell proliferation, inducing cell cycle arrest, and increasing apoptosis. These findings were in accordance with the inhibition of MEK/ERK and PI3K/AKT signaling pathways. Ruthenium complexes also led to a decrease in cellular clonogenic ability and cell migration, which was associated with the disruption of F-actin cytoskeleton integrity. Here, we demonstrated that ruthenium complexes, especially Ru7, have a high anticancer effect against CRC cells and are promising drugs to be used as a new therapeutical strategy for CRC treatment.

## 1. Introduction

Cancer is one of the leading causes of death worldwide and an important barrier to increasing average life expectancy. According to World Health Organization (WHO), it is responsible for approximately one in every six deaths, accounting for nearly 10 million deaths in 2020 all over the world [1,2]. In particular, colorectal cancer (CRC) ranks as the third most commonly diagnosed cancer in both men and women and the second in terms of higher mortality [2,3]. The current CRC treatment is based on surgery and chemotherapy, that is, 5-fluorouracil (5-FU), the most used chemotherapeutic agent [4,5]. 5-FU is an analog drug of uracil that, at the intracellular level, is converted to several active metabolites, disrupting RNA and DNA synthesis [6]. However, due to its low efficacy, it is often used in combination with platinum-based drugs [7].

Since the discovery of cisplatin in 1965 [8], the use of metal-based agents has increasingly gained interest in clinical practice for cancer treatment. Platinum(II) drugs, in particular, cisplatin, carboplatin, and oxaliplatin, exert their cytotoxic effect mainly through DNA damage induced by covalently binding and forming stable DNA adducts with guanines and adenine bases [9]. However, its irreversible binding to DNA and lack of selectivity to cancer cells, as with the drug 5-FU, results in serious side effects [7,10,11].

Ruthenium complexes have emerged as some of the most promising and attractive alternatives to traditional platinum-based compounds. They may act on different molecular targets, leading to high bioactivity and selectivity to cancer cell lines in comparison to normal cells [12,13,14,15]. In addition, some of these complexes show the ability to overcome platinum resistance [16,17,18].

Thanks to advances in research, ruthenium-based drugs have begun to be tested in preclinical and clinical development as antitumor agents. So far, three ruthenium-based complexes, imidazolium trans-[tetrachlorido(1*H*-imidazole)(*S*-dimethylsulfoxide)ruthenate(III)] (NAMI-A), imidazolium trans-[tetrachloridobis(1*H*-imidazole) ruthenate(III)] (KP1019), and [Ru(4,4′-dimethyl-2,2′-bipyridine)2(2-(2′,2″:5″,2‴-terthiophene)-imidazo[4,5-f])]Cl_2_ (TLD1433), have been investigated in phase I and II clinical trials for cancer chemotherapy. NAMI-A has shown effective anticancer activity in lung metastases [19], KP1019 and its sodium salt, KP1339, have successfully finished a phase I clinical trial for colorectal carcinoma treatment [20,21], and the ruthenium-based photosensitizer TLD1433 has completed a phase I clinical trial for the photodynamic therapy treatment of bladder cancer [22].

Organometallic ruthenium(II)−arene complexes have also demonstrated great potentialities for cancer therapy since they provide different opportunities to modulate the pharmacological properties of compounds, such as cellular accumulations and kinetic reactivities, minimizing side effects through variations in the arene and in the other coordinated ligands [23,24,25,26,27,28]. The two most studied families of these organometallic compounds are the ones bearing the Ru(η^6^-arene) [29] and Ru(η^5^-cyclopentadienyl) (Ru(η^5^-Cp)) [30,31] scaffolds. Both families have a piano-stool structure, where three of the coordination sites are occupied by (η^6^-arene) or (η^5^-Cp) ligands, which serve to stabilize the Ru(II) center, and the three remaining sites are occupied by several ligands that are able to modulate the cytotoxicity and stability of the compounds [32,33].

The first family comprises the RAPTA-type, [Ru(η^6^-arene)(PTA)X_2_] (PTA = 1,3,5-triaza-7-phosphaadamantane), and the RAED-type compounds, [Ru(η^6^-arene)(en)Cl] (en = ethylenediamine) [33]. Several of these compounds have been shown in vitro and in vivo to have anticancer and antimetastatic potential [16,34,35].

Our research group has focused on the search for new Ru(II) piano-stool cationic complexes as anticancer agents. Several complexes of the general formula [Ru^II^(η^5^-C_5_H_5_)(PP)(L)]^+^ were designed and synthesized, with PP = monodentate or bidentate phosphane and L = *N*-monodentate or *N*,*N*′/*N*,*O* bidentate heteroaromatic ligands [30,31]. Most of these compounds presented high cytotoxic activities against several human cancer cell lines (e.g., MiaPaCa, LoVo, PC3, HL-60, MCF7, HT29, A2780/A2780cisR, HeLa, MDA-MB-231, A549, NCI-H228, Calu-3, and NCI-H1975, among others), with many of them showing IC_50_ concentrations lower than those found for cisplatin [18,32,36,37,38,39,40,41].

**Figure 1:**
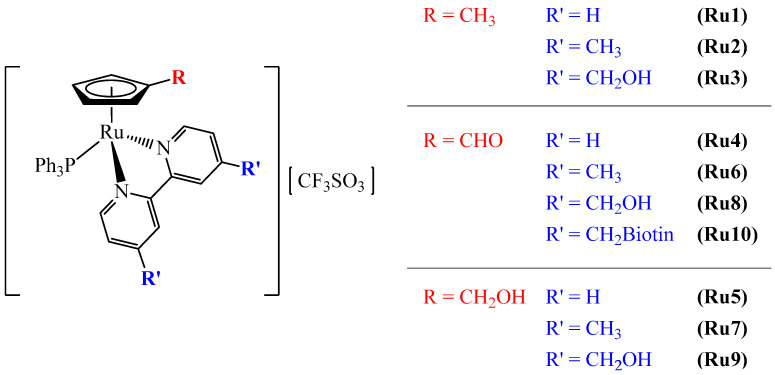
The chemical structures of ruthenium complexes Ru1 to Ru10. Ruthenium-based complexes Ru1–3 were synthesized as previously published in Côrte-Real et al. [40], and Ru4–10 in Teixeira et al. [18].

For this work, a set of ruthenium-based complexes (10 compounds) with the general formula [Ru(η^5^-C_5_H_4_R)(PPh_3_)(4,4′-R′-2,2′-bipyridine)][CF_3_SO_3_], where R = CH_3_, CHO or CH_2_OH and R′ = H, CH_3_, CH_2_OH, or dibiotin ester (Figure 1) were selected according to previous results obtained by our research group [18,40]. The inclusion of biotin has attracted much attention due to the higher tumor specificity favored by its receptor-mediated uptake. Indeed, the main biotin transporter is the sodium-dependent multivitamin transporter (SMVT), which is overexpressed in several cancer cell lines [42]. Previous studies with compounds Ru1–3 have demonstrated a very high cytotoxicity in ovarian cancer cells (A2780) and cisplatin-resistant ovarian cancer cells (A2780cisR) when compared to cisplatin [40]. Ru2 also displayed the inhibitory properties of ATP-binding cassette (ABC) pumps (MRP1 and MRP2). These efflux pumps are expressed in many human tumors, contributing to resistance to chemotherapy treatment [43]. Interestingly, the simultaneous inclusion of R = CH_3_ and R′ = dibiotin ester led to a loss of cytotoxicity with a simultaneous loss of P-gp inhibition properties by acting as a P-gp substrate [44]. Based on this, complex [Ru(η^5^-C_5_H_4_CH_3_)(PPh_3_)(4,4′-dibiotin ester-2,2′-bipyridine)][CF_3_SO_3_] was excluded in the present study. From the Ru4 to Ru10 complexes, four of them have also shown strong activity against cisplatin-resistant non-small-cell lung cancer cell lines (A549, NCI-H228, Calu-3, and NCI-H1975) and inhibited ABC pumps MRP1 and P-gp transporters [18]. Complex [Ru(η^5^-C_5_H_4_CH_2_OH)(PPh_3_)(4,4′-dibiotin ester-2,2′-bipyridine)][CF_3_SO_3_] was also excluded from the study since it was not possible to properly purify it, as previously reported by Teixeira et al. [18].

Based on the results already obtained, these compounds seem to be very promising anticancer agents. Nevertheless, there is no study on the antitumor properties of these ruthenium complexes regarding CRC. In this study, a detailed characterization of the anti-CRC properties of Ru(η^5^-Cp) complexes Ru1–10 was evaluated in two human CRC cell lines (RKO and SW480) harboring different genetic backgrounds. For this, effects on cell viability, cell cycle, apoptosis, cell migration, F-actin and on signaling pathways related to cell proliferation/apoptosis were tested.

## 2. Materials and Methods

### 2.1. Compounds under Study

All syntheses were carried out in a dinitrogen atmosphere using current Schlenk techniques, and the solvents used were dried using standard methods. All complexes had the general formula [Ru(η^5^-C_5_H_4_R)(PPh_3_)(4,4′-R′-2,2′-bipyridine)][CF_3_SO_3_], varying R and R′ substituents, where R = CH_3_, CHO or CH_2_OH and 4,4′-R′-2,2′-bipyridine = 2,2′-bipyridine (Ru1, 4, and 5), 4,4′-di(hydroxymethyl)-2,2′-bipyridine (Ru3, 6, and 7), 4,4′-dimethyl-2,2′-bipyridine (Ru2, 8, and 9), and 4,4′-dibiotin ester-2,2′-bipyridine (Ru10) (Figure 1). These compounds were synthesized using protocols recently reported by Côrte-Real et al. [40] (Ru1–3) and Teixeira et al. [18] (Ru4–10). To assess biological activities, compounds were dissolved in 100% dimethyl sulfoxide (DMSO).

### 2.2. Cell Lines and Conditions

Two human-derived colorectal cancer cell lines, RKO (BRAF^V600E^ mutation) and SW480 (KRAS^G12V^ mutation), and normal colon epithelial cells derived from the human colon, NCM460, were used in this experiment. Cell lines were maintained in T25 cm^2^ polystyrene flasks with DMEM medium for RKO and RPMI-1640 medium for SW480 and NCM460. All mediums were supplemented with 10% fetal bovine serum and 1% antibiotic-antimitotic solution. All cells were grown upon 70 to 90% confluence, then were washed with 1× phosphate buffer saline (PBS) and harvested by digestion with trypsin 0.05% (*v*/*v*). Trypsin was inactivated by complete culture media, and cells were resuspended and then transferred into new culture flasks or seeded in sterile test plates for the different assays. Cell lines were grown in cells at 37 °C, 5% CO_2_ incubator with humidified atmosphere. For all experiments, except for the colony formation and wound healing assays, RKO and SW480 cells were seeded at a density of 1 × 10^5^ cells/mL and NCM460 at 2 × 10^5^ cells/mL.

### 2.3. Cell Viability Analyzed by Sulforhodamine B Assay

Effects on cell viability and determination of IC_50_ were assessed by sulforhodamine B (SRB) assay [32]. For this, cells were seeded in 48-well plates and left to adhere for 24 h. After adhesion, cell lines were incubated with different concentrations of compounds for 48 h, starting with the maximum concentration of 100 µM and serially decreasing until 0.1 µM. For each cell line and compound, two negative controls were performed; 1st control, cells incubated only with growth medium, and 2nd control, cells exposed to growth medium with vehicle (DMSO concentration in conditions of the compounds). After a 48 h incubation, cells were fixed with ice-cold methanol containing 1% acetic acid for 90′ at −20 °C. After that, the fixing solution was removed, and the plates were left to air-dry at room temperature in the fume hood. After the fixation step, cells were stained with 0.5% (*w*/*v*) SRB dissolved in 1% acetic acid for 90′ at 37 °C protected from light. SRB dye was removed, the dye not bound was washed with 1% acetic acid, and plates were air-dried at room temperature. SRB that bonded to the cell was dissolved with 10 mM Tris pH10. Absorbance was read at 540 nm in a microplate spectrophotometer. The absorbance values measured by the microplate reader of each condition are proportional to the number of viable cells in comparison to the 2nd control, which was considered to be 100% of cell growth.

### 2.4. Cell Death Assessed by Annexin V/Propidium Iodide Assay

Induction of cell death was evaluated using annexin V/propidium iodide (AV/PI) assay and assessed by flow cytometry [45]. After 48 or 72 h incubation, cells were harvested by trypsinization, concentrated by centrifugation, and washed with PBS. The supernatant was removed via centrifugation and cells were incubated with “binding buffer”, AV, and PI (50 μg/mL), and then incubated for 15’ at room temperature in the dark. After incubation, cells were immediately analyzed by flow cytometry. Staurosporine was used as a positive control for the induction of apoptosis [46].

### 2.5. Cell Cycle Analysis by Flow Cytometry

Cells were incubated with an IC_50_ concentration of compounds and harvested 24 and 48 h later. Cells were then fixed with ice-cold ethanol (70%) for 30′ on ice, washed with PBS, and the supernatant was discarded after centrifugation. The samples were then treated with ribonuclease A (200 mg/mL) for 15′ at 37 °C and stained with PI (50 µg/mL) for 30′ in the dark at room temperature before the analysis [32]. Stained cells (20.000 single-cell events) were analyzed and sorted based on the area and pulse width of the PI signal in a flow cytometer, Beckman Coulter (Cytoflex System B4-R2-V0). Cell-cycle analyses of DNA histograms were evaluated using CytoExpert Software version 2.4 (Indianapolis, IN, USA).

### 2.6. Determination of Cell Proliferation by Carboxyfluorescein Succinimidyl Ester Labelling

Effects on cell proliferation of ruthenium-based complexes were assessed by carboxyfluorescein succinimidyl ester (CFSE) labeling by flow cytometry (Cytoflex System B4-R2-V0). Briefly, the cell suspension was concentrated by centrifugation and incubated with CFSE dye in the dark at 37 °C for 15′. After incubation, the supernatant was removed via centrifugation, and cells were washed twice with 1× PBS to remove the excess unattached dye [47]. After that, cells were plated at a density of 1 × 10^5^ cells/mL in 6-well plates and left to adhere. One day after, cells were incubated with an IC_50_ of chosen ruthenium complexes. Cells were collected at 0, 24, 48, and 72 h of incubation, and fluorescence intensity was readily analyzed with flow cytometry at an excitation/emission of 492/517 nm. Results were analyzed using CytoExpert Software 2.4.

### 2.7. Western Blot Assay

After the incubation time (48 h with the IC_50_ concentrations), cells were collected and lysed with RIPA buffer containing 20 mM of NaF, 1 mM of PMSF, 20 mM of Na_2_V_3_O_4_, and a protease inhibitor cocktail for 15′ on ice. Bio-Rad DC protein assay was used to quantify protein using BSA as standard. Protein samples (25 μg) were separated by SDS-PAGE gel electrophoresis and then semi-dry-transferred to polyvinylidene difluoride (PVDF) membranes. After protein transfer, membranes were blocked with 5% BSA in PBS with Tween-20 (PBS-T). After 3 steps of washing with PBS-T (10′ each), membranes were incubated with the primary antibodies (anti-phospho-ERK, anti-ERK, anti-phospho-AKT, anti-AKT, anti-β-Actin, and anti-GAPDH) followed by the secondary antibody conjugated with IgG horseradish peroxidase. Immunodetection was performed under the chemiluminescence detection system [48].

### 2.8. Colony Formation Assay

RKO and SW480 cells were seeded in 6-well plates at a density of 300 cells/mL and 500 cells/mL, respectively. After a 24 h plating, cells were incubated with ¼, ½, and IC_50_ concentrations of the different complexes. Medium with compounds was removed 48 h after the incubation and renewed every 3 days. Colonies were monitored by microscopy every two and two days. The assay was ended when more than 50 cells per colony were achieved. Wells were washed with PBS and stained with a solution of 6% (*v*/*v*) glutaraldehyde with 0.5% (*w*/*v*) crystal violet for 1 h. The plate was washed with fresh water and left to air-dry. Colonies were counted using ImageJ software. The negative control was incubated with the concentration of the vehicle [45].

### 2.9. Wound Healing Assay

The wound healing assay was performed in 6-well plates coated with a confluent monolayer of cells. This protocol was adapted from Liang et al. [49]. For this, cell lines were seeded at a density of 5 × 10^5^ cells/mL and left to adhere for 24 h. After achieving a confluent monolayer, a ‘‘scratch’’ was created using a p20 pipet tip. Cell debris and cells in suspension were removed by washing the wells twice with 1× of PBS and then a growth medium with compounds was added. Cell lines were incubated for 12 and 24 h with IC_50_ concentrations and 2× IC_50_ concentrations of the different compounds. For each condition, 4 photographs were taken with a microscope at matching reference points. Images were acquired at 0, 12, and 24 h, and wound closure was evaluated using the ImageJ software.

### 2.10. Effect on F-Actin by Phalloidin Staining

Effects on the cytoskeleton, or, more specifically, in actin fibers, were assessed by phalloidin staining using fluorescence microscope visualization in an inverted microscope (Olympus BX63 F2,Tokyo, Japan). Cells were seeded in coverslips into a well and allowed to attach for 24 h. Cells were then treated with the IC_50_ of each compound for 48 h. After the incubation time, wells were washed with warmed PBS and fixed for 20′ in 4% paraformaldehyde. After that, coverslips were removed from the well, washed with PBS, and permeabilized with 0.1% Triton X-100 for 5′. After another washing step, cells were incubated in the dark with Phalloidin-Alexa Fluor^®^ 568 for 2 h. The nucleus was stained with DAPI in Vectashield after washing steps. Coverslips were inverted onto the slides and stored at 4 °C in the dark until analysis [46].

### 2.11. Statistical Analysis

Statistical analysis was performed with the Graph Pad Prism 5 Software (Graphpad Software, San Diego, CA, USA). Data are expressed as mean ± standard deviation (SD) from at least three independent experiments. One-way ANOVA followed by the Bonferroni test was used to assess differences among groups. Values were considered statistically different for *p*-values < 0.05 for a confidence level of 95%.

## 3. Results

### 3.1. Ruthenium Complexes Affect Cell Viability at Low Doses

The effects on the cell viability of the ruthenium complexes were assessed with an SRB assay in RKO and SW480 cell lines. The study of the effects on cell viability began with screening the compounds that showed an effect, with a maximum dose of 100 µM. Compounds where the maximum dose tested did not achieve 50% inhibition were removed from the study. A dose-dependent decrease in cell viability in both cell lines, RKO and SW480, was observed after 48 h of incubation with the compounds (Figures S1 and S2). The half-maximal inhibitory concentration (IC_50_) values of complexes are listed in Table 1. Of the ten complexes tested, three in the SW480 cell line (Ru8–10) and two in the RKO (Ru8 and Ru10) presented IC_50_ concentrations above 100 μM. The remaining ruthenium complexes displayed low viability at relatively low doses. Overall, RKO cells were more sensitive than SW480, showing lower IC_50_ concentrations. In both the SW480 and RKO cells, complexes Ru1–7 demonstrated a higher inhibitory effect on cell viability than the chemotherapy drugs cisplatin and 5-FU, except for Ru4 in SW480.

**Figure 2:**
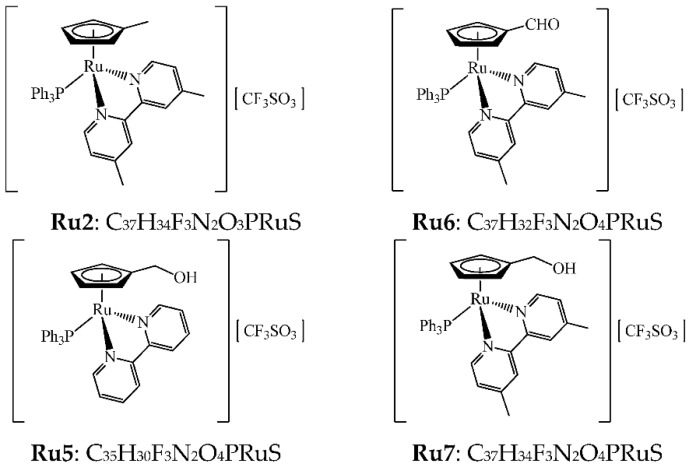
Chemical structures of ruthenium-based complexes selected for further studies.

According to their lower IC_50_ concentrations, four complexes were chosen to pursue studies (Ru2, 5–7; Figure 2). The IC_50_ concentrations of the chosen compounds were also assessed in the NCM460 cell line (normal colon epithelial cells). Higher IC_50_ concentrations were observed in NCM460 cells in comparison to colorectal cancer cells, RKO, which were translated into a higher selectivity index of the complexes in this CRC cell line. In SW480, a lower selectivity was found, with the complex Ru7 being the most selective.

### 3.2. Ruthenium Complexes Induce Morphological Changes in Cells

**Figure 3:**
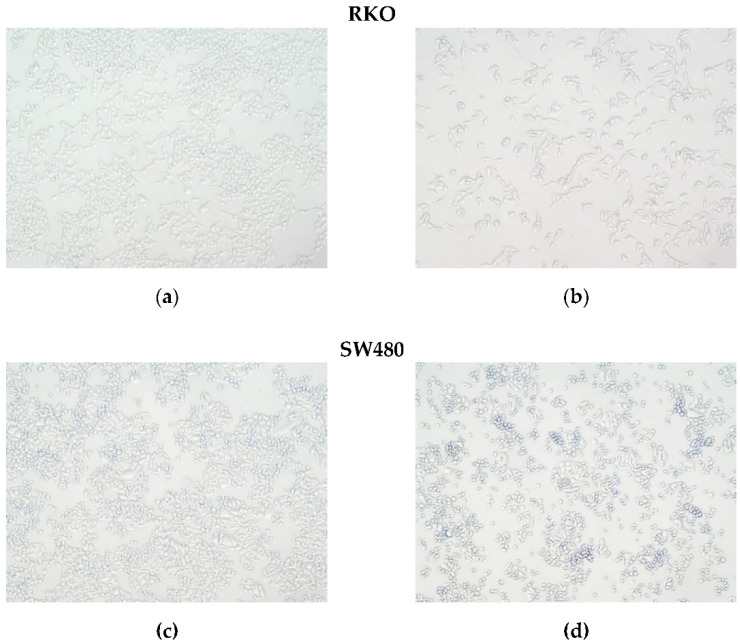
Morphological changes in RKO and SW480 cells assessed by microscopy (100×) upon 48 h incubation with an IC_50_ value of Ru2. (**a**) Normal morphology of RKO, (**b**) RKO cells incubated with Ru2, (**c**) normal morphology of SW480, and (**d**) SW480 cells incubated with Ru2. Similar effects were found with remaining complexes (data not shown).

The effects of ruthenium complexes on cell morphology after treatment with IC_50_ for 48 h were examined by phase contrast in the RKO and SW480 cells (Figure 3). The control group showed its normal shape with intact membrane integrity. In cells treated with ruthenium complexes, a significant decrease in the number of adherent cells and cell shrinkage was observed in both cell lines. In SW480, a higher number of cells in suspension was observed.

### 3.3. Ruthenium Complexes Induce Apoptosis

**Figure 4:**
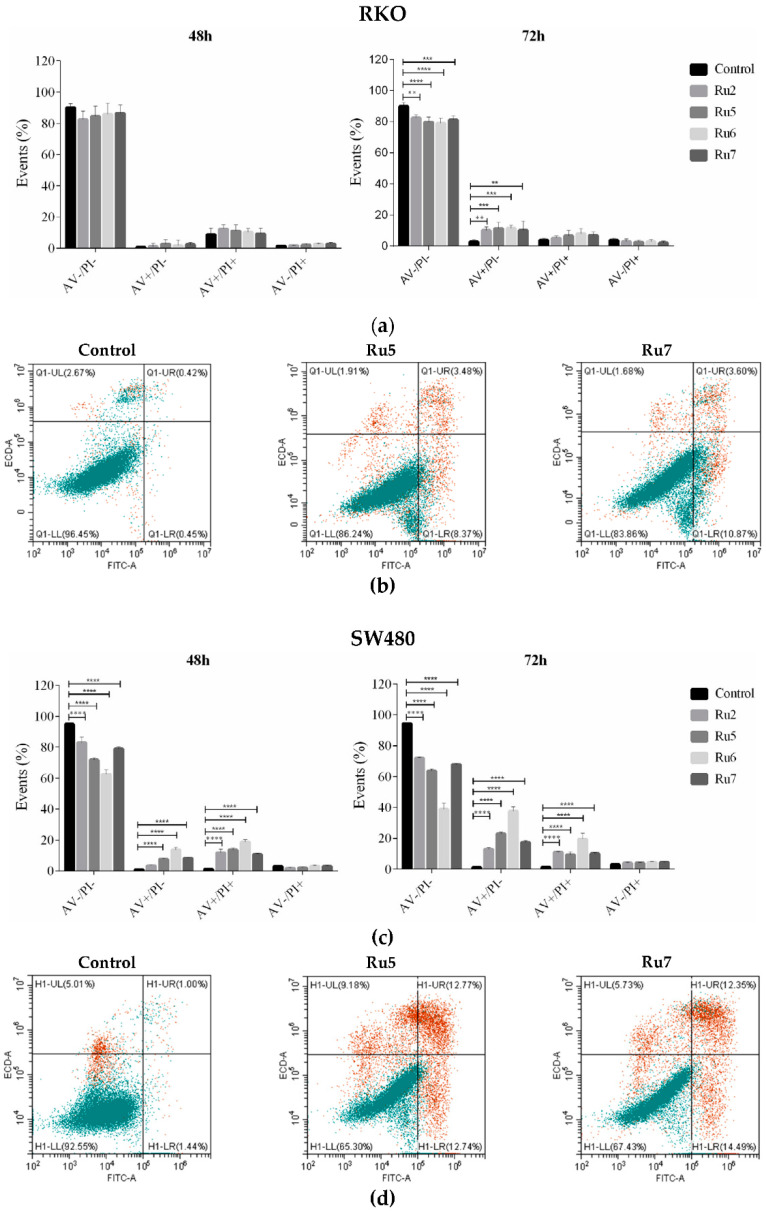
The effects of ruthenium complexes on the induction of cell death after 48 and 72 h of incubation with IC_50_ concentrations in the RKO (**a**) and SW480 (**c**) cell lines. Dot plot diagrams obtained with flow cytometry of the RKO (**b**) and SW480 (**d**) cells treated with an IC_50_ concentration of Ru5 and Ru7 for 72 h after dual staining with Annexin V-FITC and PI. Data are presented as mean ± SD from at least three independent experiments. Results were statistically different from the negative control for ** *p* < 0.01, *** *p* < 0.001 and **** *p* < 0.0001.

The cell death mechanism was assessed using AV/PI with a cytometry-based assay. The RKO and SW480 cell lines were incubated with the ruthenium complexes for 48 and 72 h with their respective IC_50_ concentrations. The results show that all the ruthenium complexes led to an overall increase in the percentage of AV-positive stained cells, AV+/PI− (early apoptosis) and AV+/PI+ (late apoptosis) cells, in comparison to the negative control, especially in SW480 cells after 72 h incubation (Figure 4). PI is a nuclear dye that stains both late apoptotic and necrotic cells in which cells membrane permeability is compromised. AV is a marker of both early and late apoptosis since it binds the phosphatidylserine that is translocated to the external cellular membrane leaflet during apoptosis. AV is widely used in conjunction with PI to differentiate between early apoptosis (AV+/PI−), late apoptosis (AV+/PI+), and necrosis (AV−/PI+) through differences in plasma membrane integrity and permeability. Based on the results obtained with this assay, we conclude that these compounds induced cell death by apoptosis. In the RKO cell line, after 48 h of incubation, the induction of apoptosis was not significantly different in comparison with the control; nevertheless, it increased significantly after 72 h of incubation, where a higher number of AV positive cells (AV+/PI−) were observed. In the SW480 cells, a significant increase in AV+ cells was observed at 48 and 72 h of incubation. This increase was particularly evident in the compound Ru6 since it was the most effective compound in inducing apoptosis at both the early and late stages, especially in the SW480 cells. The incubation with our complexes did not show increase PI-positive cells, which is indicative that these drugs do not induce necrosis.

### 3.4. Ruthenium Complexes Induce Cell Cycle Arrest

**Figure 5:**
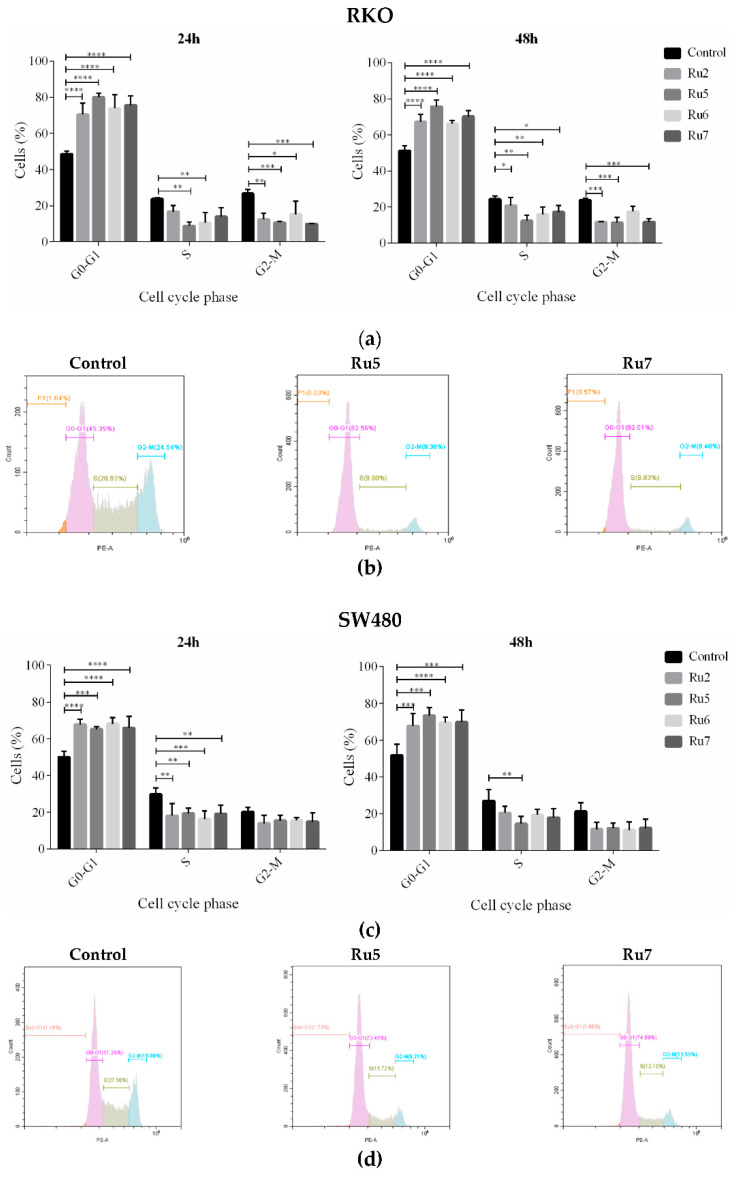
Analysis of cell-cycle phase distribution after 24 and 48 h of incubation with IC_50_ concentrations in RKO (**a**) and SW480 (**c**) cell lines. Flow cytometry histograms of the FL2 red channel reading fluorescence of RKO (**b**) and SW480 (**d**) cells incubated with IC_50_ concentrations of Ru5 and Ru7 complexes for 48 h and stained with propidium iodide. Results were expressed as mean ± SD of at least three independent experiments. Results were statistically different from the negative control for * *p* < 0.05, ** *p* < 0.01, *** *p* < 0.001 and **** *p* < 0.0001.

The effects of new ruthenium complexes on the cell cycle were analyzed through PI-staining using flow cytometry. The distribution of cells in the different cell cycle phases is displayed in Figure 5. After a treatment with an IC_50_ concentration of the compounds, a clear accumulation in the proportion of cells in the G0/G1 phase of the cell cycle was observed in both CRC cells lines after 24 and 48 h of incubation. This led to a corresponding reduction in the percentages of cells in the S and G2/M phases. Cell distribution analysis showed that treatment with Ru5 gave rise to the best effect in RKO at both 24 and 48 h of incubation, and in SW480 at 48 h of incubation.

### 3.5. Ruthenium Complexes Inhibit Cell Proliferation

**Figure 6:**
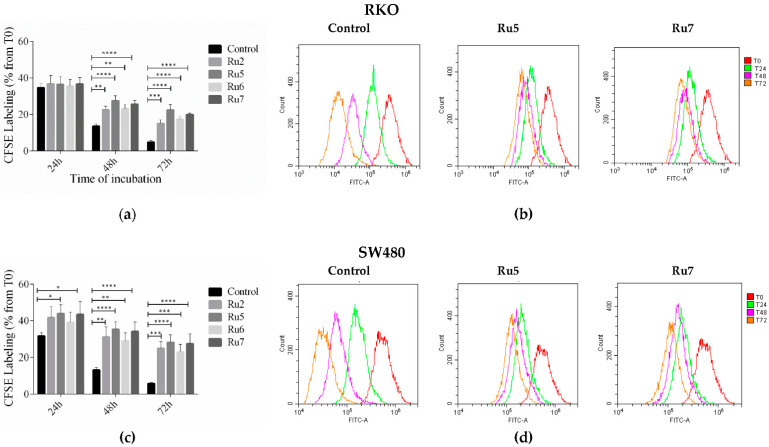
The effect of ruthenium complexes on cell proliferation assessed by CFSE labeling after 24, 48, and 72 h of incubation with an IC_50_ concentration in the RKO (**a**) and SW480 (**c**) cell lines. A CFSE histogram profile of the RKO (**b**) and SW480 (**d**) cells treated with Ru5 and Ru7 for 24, 48, and 72h. Results were statistically different from the negative control for * *p* < 0.05, ** *p* < 0.01, *** *p* < 0.001 and **** *p* < 0.0001.

Since we observed a cell cycle arrest in G0/G1, and to further confirm these results, an evaluation of cell proliferation was performed by CFSE labeling over a 3-day period. Effects on cell division/proliferation were evaluated by the CFSE fluorescence intensity using flow cytometry. In control cells, fluorescent intensity decreased linearly over time. In both cell lines, incubation with IC_50_ of ruthenium complexes induced a decrease in cell division shown by the smallest decrease in intensity of cell stained with the probe. In RKO cells, statistical differences were observed after 48 and 72 h incubation, whereas in SW480 were also observed after 24 h incubation with Ru5 and Ru7 (Figure 6).

### 3.6. Effect on Signaling Pathways Related with Cellular Survival

**Figure 7:**
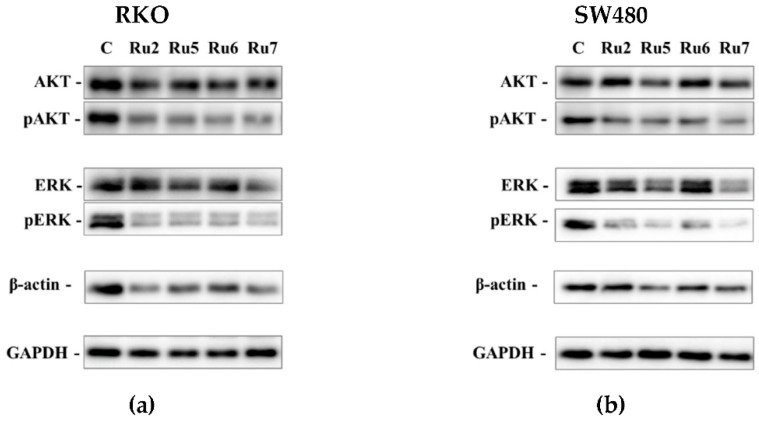
Western blotting of the RKO (**a**) and SW480 (**b**) cell lines for AKT, pAKT, ERK, pERK, β-actin, and GAPDH after 48 h of incubation with an IC_50_ of ruthenium complexes.

A Western blot analysis of the pERK/ERK and pAKT/AKT expression levels was assessed to better elucidate the effects of the complexes on signaling pathways related to proliferation, growth, and survival (MEK/ERK and PI3K-AKT). GAPDH was used as a housekeeping protein for the comparison of protein expression levels. β-actin expression, a major component of the cytoskeleton involved in cell migration and apoptosis, was also analyzed. In both cell lines, treatment with Ru2, 5–7 induced downregulation in both the MEK/ERK and PI3K-AKT signaling pathways, demonstrated by a decrease in phosphorylated AKT and ERK and not the total AKT and ERK when compared to the GAPDH protein control (Figure 7). The expression of β-actin was also downregulated in cells incubated with ruthenium complexes when compared to GAPDH control.

### 3.7. Ruthenium Complexes Inhibit Clonogenic Ability

**Figure 8:**
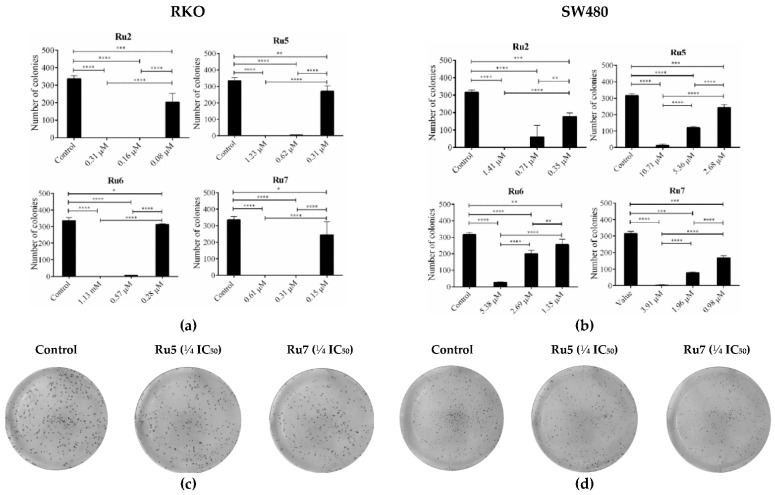
Colony formation ability after 48 h of incubation with IC_50_, ½ IC_50_, and ¼ IC_50_ of ruthenium complexes in the RKO (**a**) and SW480 (**b**) cell lines and the representative images of colony formation in the RKO (**c**) and SW480 (**d**) cell lines after incubation with Ru5 and Ru7 complexes. Data are presented as mean ± SD from at least three independent experiments. Results were statistically different from the negative control for * *p* < 0.05, ** *p* < 0.01, *** *p* < 0.001 and **** *p* < 0.0001.

To evaluate the effect of ruthenium complexes on cellular clonogenic ability (number of colonies), we treated the SW480 and RKO colorectal cancer cells with the most promising complexes, Ru2, 5–7. We started the experiments by evaluating the effects of incubation with IC_50_ concentrations for 48 h. The concentrations were reduced until ¼ IC_50_, where was possible to observe the formation of colonies. In both CRC cell lines, all of the complexes were able to reduce the ability of a cell to proliferate indefinitely, thereby diminishing its replicative potential to form a large colony or a clone (clonogenic ability) (Figure 8). In RKO cells incubated with IC_50_ concentration, no colony formation was observed for any of the complexes, and a very low number of colonies were presented at ½ IC_50_ concentrations. Ru2 and Ru7 were the most effective complexes with respect to inhibiting colony formation, displaying the lowest number of colonies after incubation with ¼ IC_50_ concentrations. In the SW480 cell lines, a very low number of cells were able to recover and form colonies in ¼ IC_50_ concentration.

### 3.8. Ruthenium Complexes Inhibit Cell Migration

**Figure 9:**
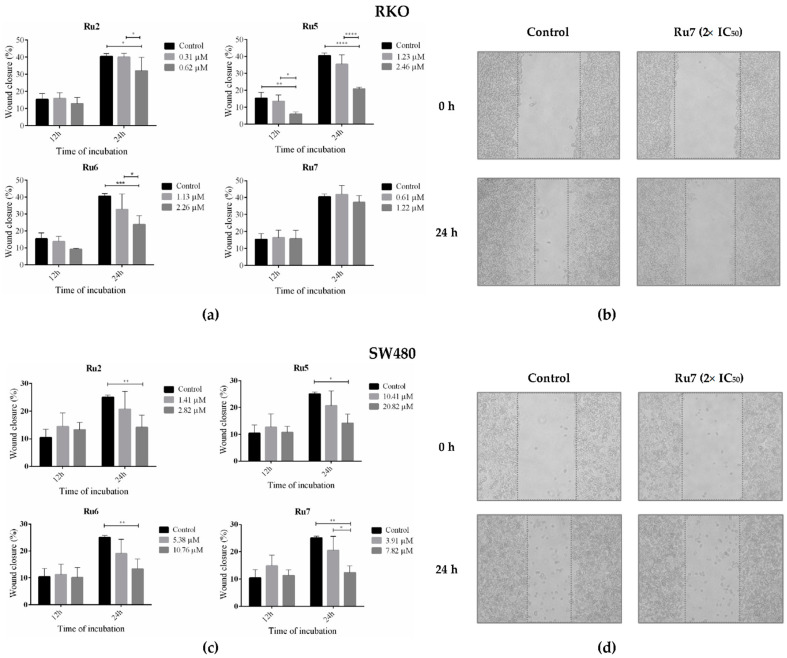
Quantitative analyses of wound closure (%) the RKO (**a**) and SW480 (**c**) cell lines incubated for 12 and 24 h with IC_50_ and 2× IC_50_ with Ru2, 5–7. Wound closure was calculated from the area in time 0 h. Representative images (100×) of the wound-healing assay of the RKO (**b**) and SW480 (**d**) cell lines after incubation with 2× IC_50_ for 0 and 24 h. Data are presented as mean ± SD from at least three independent experiments. Results were statistically different from the negative control for * *p* < 0.05, ** *p* < 0.01, *** *p* < 0.001 and **** *p* < 0.0001.

To understand if ruthenium complexes have an influence on cell migration, a wound healing assay was performed by exposing both colorectal cancer cell lines to the IC_50_ and 2× IC_50_ concentrations of complexes Ru2, 5–7 for 12 and 24 h. In the control conditions, the cells were exposed to the vehicle control (0.1% DMSO). After the incubation with the ruthenium complexes, the wound closure was evaluated for control cells and for incubated cells. In the control condition of RKO cells, a wound closure of 15 and 40% was observed at 12 and 24 h, respectively. The wound closure in SW480 cells was lower, being 10% at 12 h and 25% at 24 h (Figure 9). In both cell lines, incubation with 2× IC_50_ induced a significant decrease in cell migration at 24 h of incubation, except with Ru7 in the RKO cells.

### 3.9. Ruthenium Complexes Change F-Actin Cytoskeleton Structure

**Figure 10:**
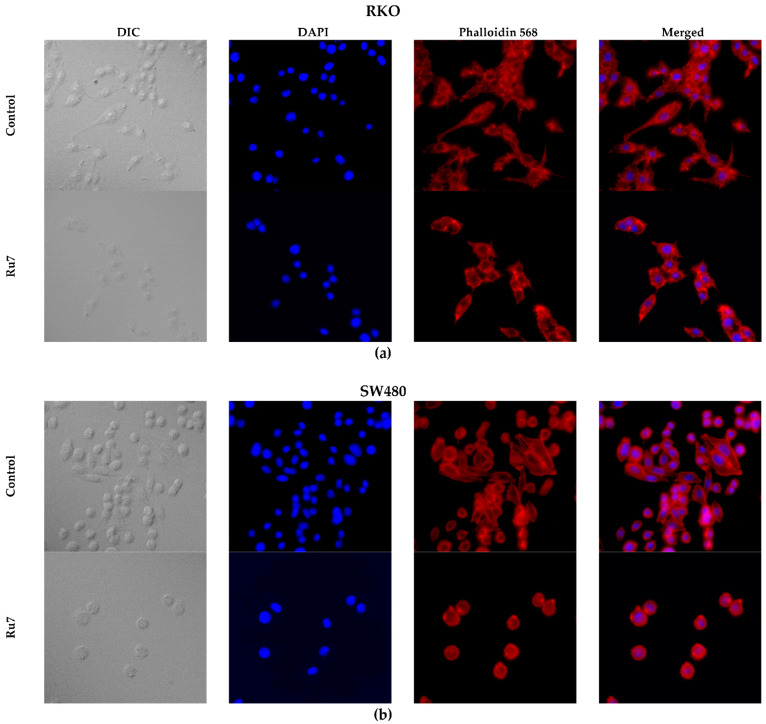
Morphological changes in the cytoskeletons of the RKO (**a**) and SW480 (**b**) cells after incubation with Ru7 for 48 h. Representative images (400×) were obtained in a fluorescence microscope. Pictures of differential interference contrast (DIC), nuclei stained with DAPI (blue), F-actin-stained with Phalloidin-Alexa Fluor^®^ 568 (red) and merged. Similar effects were found with the remaining complexes (data not shown).

Aiming to evaluate the effects of our ruthenium complexes in the cytoskeleton structure and cell morphology, we performed rhodamine-phalloidin and DAPI staining in both colorectal cancer cells. The cells were treated with IC_50_ concentrations of each compound for 48 h. Rhodamine-phalloidin binds specifically to F-actin (a structural filamentous unit of the cytoskeleton), whereas DAPI in Vectashield specifically binds to DNA present in the nucleus. In the negative control, we observed that cells are larger, with a well-marked and enlarged cytoskeleton around the nucleus (Figure 10). We could also distinguish the establishment of connections between cells showing filipodia-like structures. In the conditions treated with ruthenium complexes, most of the nuclei appeared intact and spherical; however, we also observed cell shrinkage, loss of the filipodia-like structures, and a decrease in the number of cells per condition. Effects on cell morphology were more evident in the SW480 cells, where a marked cell roundness and shrinkage, as well as a loss of cell-to-cell adhesion and intercellular communication were observed.

## 4. Discussion

Colorectal cancer is one of the most common cancers worldwide, presenting high mortality in advanced cases [2]. Currently, there are limited therapeutic drugs available for CRC therapy, for example, 5-FU, the most used chemotherapeutic agent [4,5]. Due to its low efficacy, 5-FU is often used in combination with platinum-based drugs [7]. Many platinum drugs, such as cisplatin, carboplatin, and oxaliplatin, are applied in chemotherapy regimens for the treatment of CRC and many other types of cancer. Nevertheless, cancer cells develop resistance against platinum drugs through cellular self-defense systems, such as mechanisms that reduce cellular drug accumulation, increase the detoxification system, increase DNA repair process, and decrease apoptosis and autophagy [50]. In addition, these compounds also induce several side effects due to their non-specificity to cancer cells [7,10]. The antitumor activities of different metal-based compounds have been investigated due to their potential as alternatives to platinum drugs. So far, ruthenium-based complexes have emerged as the most promising metal complexes for cancer treatment, presenting several advantages such as higher specificity and lower cytotoxicity [11,12,51].

Ruthenium complexes with the general formula [Ru(η^5^-C_5_H_4_R)(PPh_3_)(4,4′-R′-2,2′-bipyridine)][CF_3_SO_3_] (R being CH_3_, CHO or CH_2_OH and R′ being H, CH_3_, CH_2_OH, or dibiotin ester) were investigated as anti-CRC drugs by analyzing their activity, as well as by evaluating their mechanism of action.

Our results show that our set of ruthenium complexes (Ru1–10) was at least three-fold more active against the RKO cells than in the SW480 cells. Remarkably, Ru1–7 displayed lower IC_50_ than the cisplatin and 5-FU chemotherapeutic drugs in both CRC cell lines, with the exception of Ru4 in the SW480 cell line. A similar range of IC_50_ concentrations was previously obtained in ovarian cancer cell lines (A2780 and A2780cisR) for compounds Ru1–3 [40] and in non-small-cell lung cancer cell lines (A549, NCI-H228, Calu-3, and NCI-H1975) for compounds Ru4–7 [18].

These ruthenium(II)–cyclopentadienyl-derived complexes also presented selectivity against CRC, especially RKO cells, in comparison to noncancerous cells, which is a highly essential and promising feature. According to Lica et al. [52], the higher the selectivity index of a drug is, the more suitable it is for human clinical uses, with values higher than one indicating suitable selectivity against cancer cells. Other researchers have reported that compounds are selective at values greater than two [53], and in other studies, it is classified as remarkable (SI above 12), moderate (from 6 to 12), and weak (from 1 to 5) [54]. In the RKO cell line, Ru2, 5–7 complexes displayed the highest selectivity index, ranging from 4.42 to 12.20, with Ru5 and Ru7 being the most selective. This result is of major importance since the mutation in BRAF present in RKO cells, although present in only 10% of CRC patients, is associated with poor prognostic factors in advanced metastatic colorectal cancer [55]. In SW480 cells, harboring a KRAS mutation, the selectivity index was lower (1.04–2.06), being that the complex Ru7 displayed the best SI (2.06). These results are a good starting point since it is expected that, during a chemotherapy cycle, the dosage of a drug should kill cancer cells without affecting the normal cells of the organism. One of the mainstays and goals of clinical oncology has been the development of therapies promoting the effective elimination of cancer cells. Although tumor cell death by apoptosis or necrosis results in response to therapy, the cell death mechanism is of major importance. Apoptosis is a programmed cell death process that is mediated by several signaling pathways triggered by multiple factors (including cellular stress, DNA damage, and immune surveillance) that culminate in selective and controlled cell death, which is the preferred effect of an anticancer agent [56]. On another hand, necrosis is a non-programmed and not selective form of cell death, that affects normal cells and leads to inflammatory diseases and processes [57].

Treatment with IC_50_ concentrations of ruthenium(II)-based compounds induced changes in cell morphology, a reduction in cell numbers, increased cells in suspension, and cell shrinkage. Based on these observations, the type of cell death induced by these compounds was evaluated. In both cell lines, incubation with ruthenium complexes resulted in an overall increase in both early apoptosis and late apoptosis (increased AV positive population when compared to the control) after 72 h of incubation. This increase was particularly evident in the SW480 cells (two-fold higher than in the RKO cells) harboring a TP53 mutation. In addition, ruthenium complexes did not result in the induction of necrosis, as indicated by the percentage of PI-positive cells. It is known that p53 status plays an important role in sensitivity to chemotherapeutic drugs by inducing programmed death [58]. However, in both cell lines, the p53 status did not seem to change the response to the compounds, as the induction of apoptosis was higher in SW480 (TP53-mutated) when compared to RKO (TP53 wild type). These results were previously reported with respect to HCT116 (TP53 wild type) and HCT116 (TP53-null), where the induction of apoptosis independently of p53 status was also observed after treatment with several ruthenium compounds: [Ru(η^6^-*p*-cym)(*p*-Impy−NMe_2_)I]PF_6_ (Impy–imino-pyridine; NMe–*N* methyl) [59]; ruthenium complexes [Ru(η^6^-flu)(en)Cl]^+^ (flu = fluorene) and [Ru(η^6^-dihyphen)(en)Cl]^+^ (dihyphen being 9,10-dihydrophenanthrene) [60]; and [Ru(η^6^-*p*-cym)(CipA-H)Cl] (CipA being 7-(4-(Decanoyl)piperazin-1-yl)-ciprofloxacin) [61].

Cancer is frequently considered a cell cycle disease due to alterations in different cell-cycle regulators, which play an important role in tumor development [62]. The inhibition of cell cycle progression in cancer cells is, therefore, an important strategy in controlling cancer progression. In accordance with this, cell-cycle analyses show that all ruthenium complexes lead to an increase in the percentage of cells in the G0/G1 phase. The effects on the cell cycle were more evident in the RKO cells than in the SW480 cells, where increases of 60 and 40% were, respectively, reached.

The effect of ruthenium complexes on cell cycle progression, proliferation, and apoptosis led to the analysis of some proteins involved in pathways that regulate those phenotypic alterations. The MEK/ERK and PI3K/AKT signaling pathways are the key cell signaling pathways involved in the cell cycle progression from the G1 to the S phase, cell proliferation, survival, apoptosis, and tumorigenesis [62]. Both cell lines have the activation of MEK/ERK and PI3K/AKT signaling pathways through BRAF^V600E^ and PIK3CA mutations in RKO and KRAS^G12V^ mutation in SW480 cells. Incubation with ruthenium complexes was shown to inhibit the activation of both MEK/ERK and PI3K/AKT signaling pathways via the downregulation of pAKT and pERK when compared to the control. These results are in corroboration with the cell cycle arrest results (arrest at the G0/G1 phase), decreased cell proliferation assessed by CFSE assay, and an increase in apoptosis via AV/PI assay.

One of the main flaws in chemotherapy regimens is that some cells can relapse and maintain their malignant potential even after several cycles of chemotherapy. In this way, the effects of ruthenium-based complexes on cell survival and proliferation were assessed via colony formation assay. This assay allows us to determine a cell’s ability to survive exposure to an exogenous agent and to produce colonies after that agent is removed, simulating in vitro what happens during cycles of chemotherapy [46].

Our results show that, in cells exposed to IC_50_ concentrations for 48 h, and more than one week after the removal of the agents, no RKO cells were able to grow and form colonies, while in SW480 cells, only a few colonies were formed. To observe colonies, we reduced the concentration of the compounds to ¼ IC_50_ concentrations. In these conditions, all the compounds significantly reduced the clonogenic ability (number of colonies) of the cells. Overall, the results presented herein are very exciting in regard to the possible application of these new ruthenium complexes in chemotherapy, since the number of cells that may relapse might be significantly reduced in low concentrations.

Cell migration and invasion are central points of the pathological and physiological phenomena of metastatic cancer. The wound healing assay can be used as a predictive method to study the inhibitory potential of anticancer drugs on cell migration, which is a relevant feature of cancer cell metastatic ability [63]. An overall decrease in wound closure percentage was observed at 24 h of incubation with ruthenium complexes, suggesting that ruthenium complexes may inhibit cell motility capacity, thus inhibiting cell migration. Moreover, the identification of effective compounds able to interfere with cancer cell migration, and, possibly, invasion, is of the utmost importance in preventing metastatic dissemination in the malignant progression of cancer [64,65].

Motivated by the results obtained, we further investigated the effects on cytoskeleton architecture and assessed the nuclear integrity of cancer cell lines upon treatment with ruthenium complexes using rhodamine-phalloidin and DAPI staining. In cells treated with ruthenium complexes, disruption in the normal cytoskeleton architecture was observed. Although most of the nuclei appeared intact and spherical, cell shrinkage, cell adhesion destruction, and loss of filipodia-like structures were observed after incubation with ruthenium complexes. In addition, we observed a decreased cell number, which leads to an increased distance between cells, affecting cell–cell adhesion and intercellular contact establishment, thus decreasing the communication between cells. The cytoskeleton is responsible for resisting changes in cell morphology, spatially organizing the cellular organelle, and supporting the appropriate functioning of the cells, as well as cell motility, migration, and apoptosis. Specifically, the motility of cells is organized by the polymerization and cross-linking of cytoskeleton proteins, such as actin filaments conducting the formation of filopodia and lamellipodia during tumor cell migration [66,67]. The results obtained from the cytoskeleton architecture analysis are in accordance with the decrease in β-actin expression and support the inhibition of migration induced by ruthenium complexes. Moreover, most apoptotic pathways involve the activation of caspases, and the cytoskeleton may be a target for the proteolytic activity of some caspase downstream effectors. Thus, morphological changes and alterations in the actin cytoskeleton organization can be observed in apoptotic cells [68,69]. Changes in cytoskeleton architecture were particularly evident in the SW480 cells, where the induction of apoptosis was higher. These changes in cell morphology and the organization patterns of the actin cytoskeleton have already been reported in lung cancer cells (A549) treated with ruthenium (II) complex cis-[Ru(3-hydroxy-4-methoxybenzoate)(bis(diphenylphosphino)methane)_2_][PF_6_] [70].

## 5. Conclusions

The novel ruthenium complexes were found to be very active and specific even at low concentrations in colorectal cancer cell lines and more selective for CRC cells than for noncancer cells, particularly Ru7. These novel complexes displayed an ability to induce cell cycle arrest at the G0/G1 stage, to decrease cell proliferation, and to induce cell death by apoptosis via the downregulation of MEK/ERK and PI3K/AKT signaling pathways. They were able to significantly reduce cell migration and the clonogenic ability of the cells. In addition, ruthenium complexes disrupted actin cytoskeleton integrity which correlates with the decrease in motility and induction of apoptosis. In the RKO cell line, ruthenium complexes seem to have more effects on inhibiting proliferation, whereas in SW480, ruthenium complexes seem to be more prone to inducing apoptosis. Taken together, these results highlight the potential use of ruthenium complexes as anti-CRC drugs, providing opportunities to develop new therapeutic approaches for CRC. Ruthenium complexes open exciting possibilities to overcome some of the major obstacles in CRC treatment, such as low selectivity toward cancer cells, which is associated with general toxicity, side effects, and drug resistance. Summing up, ruthenium(II)–cyclopentadienyl–organometallic compounds are new and promising anticancer drugs for CRC therapy.

## Figures and Tables

**Table 1:** IC_50_ concentrations determined after 48 h of incubation of ruthenium complexes in two colorectal cancer cell lines (RKO and SW480) and a normal colon cell line (NCM480). Selectivity index (SI) was evaluated in CRC cells compared with NCM460.

	IC_50_ (µM)			SI	
Compounds	RKO	SW480	NCM460	RKO	SW480
Ru1	0.54 ± 0.06	2.01 ± 0.18			
Ru2	0.31 ± 0.04	1.41 ± 0.11	1.46 ± 0.14	4.42	1.04
Ru3	2.75 ± 0.15	8.15 ± 0.24			
Ru4	4.13 ± 0.24	26.53 ± 1.12			
Ru5	1.23 ± 0.08	10.71 ± 0.18	13.83 ± 0.54	11.24	1.29
Ru6	1.12 ± 0.12	5.38 ± 0.42	8.47 ± 0.57	7.50	1.57
Ru7	0.61 ± 0.07	3.91 ± 0.32	7.44 ± 1.31	12.20	2.06
Ru8	>100	>100			
Ru9	45.25 ± 2.09	>100			
Ru10	>100	>100			
5-FU	4.85 ± 0.09	46.68 ± 0.15			
Cisp	16.03 ± 0.07	19.92 ± 0.06			

Values expressed as mean ± SD.

## Data Availability

Data is contained within the article and supplementary material.

## Supplementary Materials

The following supporting information can be downloaded at: https://www.mdpi.com/article/10.3390/pharmaceutics14061293/s1, Figure S1. The dose–response curves of cell viability in the RKO cell line shown for the ruthenium complexes after 48 h incubation. Figure S2. The dose–response curves of cell viability in the SW480 cell line shown for the ruthenium complexes after 48 h incubation.
