# Supplementary material for: Ruthenium(II)–Cyclopentadienyl-Derived Complexes as New Emerging Anti-Colorectal Cancer Drugs
Source: Pharmaceutics. 2022 Jun 17;14(6):1293. doi: 10.3390/pharmaceutics14061293 (PMC9228117; doi:10.3390/pharmaceutics14061293)
Supplement: Supplementary file 1 [file pharmaceutics-14-01293-s001.zip › pharmaceutics-1755028-supplementary.pdf]

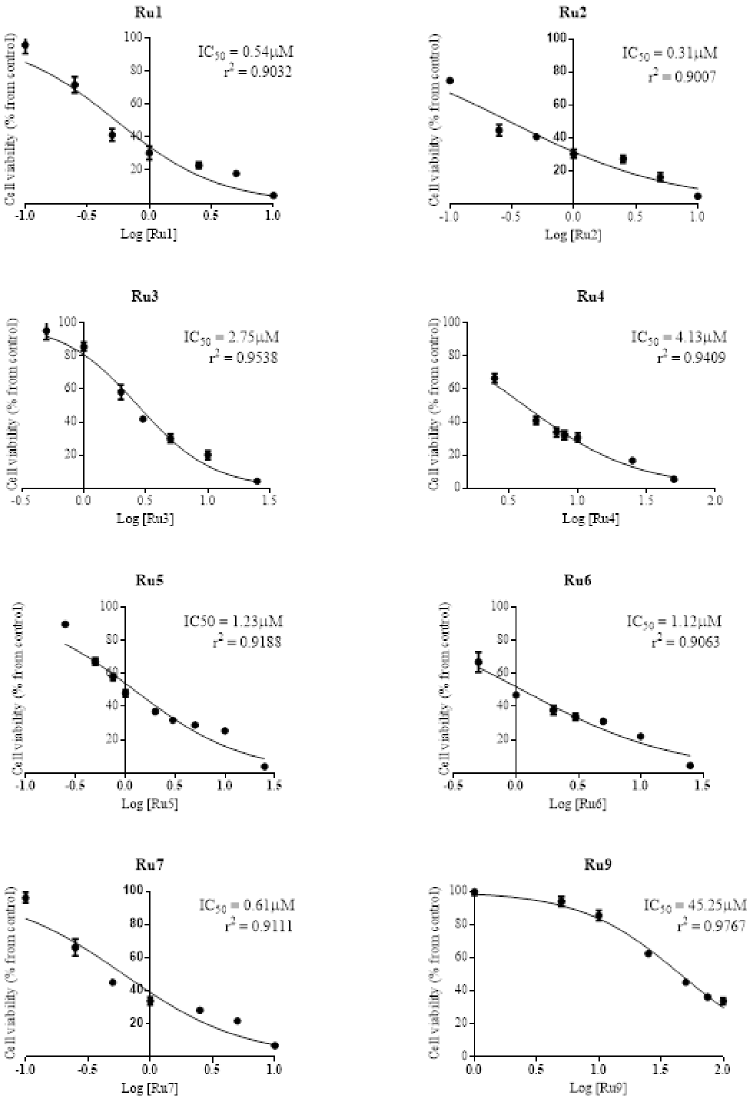

Figure S1. Dose-response curves of cell viability in RKO cell line shown for the ruthenium complexes after 48 h incubation.

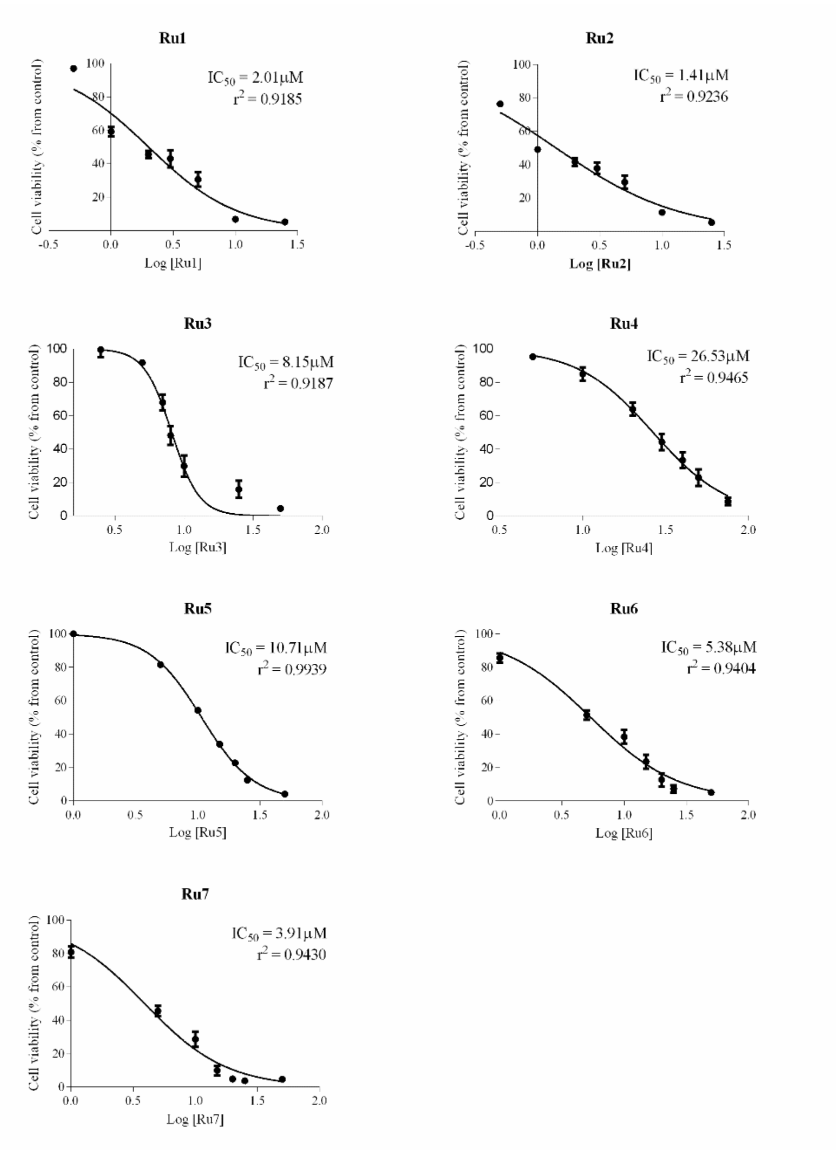

Figure S2. Dose-response curves of cell viability in SW480 cell line shown for the ruthenium complexes after 48 h incubation.
